# A Narrative Review on Intermittent Fasting as an Approachable Measure for Weight Reduction and Obesity Management

**DOI:** 10.7759/cureus.30372

**Published:** 2022-10-17

**Authors:** Raghav Janaswamy, Pallavi Yelne

**Affiliations:** 1 Medicine, Jawaharlal Nehru Medical College, Datta Meghe Institute of Medical Sciences, Wardha, IND

**Keywords:** time restricted feeding, intermittent calorie restriction, intermittent fasting, diabetes mellitus type 2, obesity

## Abstract

Obesity can be regarded as the curse of this modern advanced and efficient lifestyle as it is the crux of very precarious comorbidities. The prevalence of obesity is so widespread that cases of obesity can be seen on either end of the age spectrum. With the rise of the twenty-first century and the rise of ease of living, the sedentary lifestyle also went on the rise to become the primary contributor to the rise in obesity. For the management of obesity, various dietary modifications grew in popularity, among which is intermittent fasting. Intermittent fasting grew in popularity with the rise of the internet. Intermittent calorie restriction/time-restricted feeding is a form of caloric restriction revolving around a short window for eating and a comparatively larger window for fasting. This form of feed-fast cycle promotes increased consumption of adipose tissue and glycogen stores, leading to increased fat loss and reduced satiety. Intermittent fasting is also said to have cardioprotective functions as well known to control diabetic parameters and reduce the incidence of diabetes. This narrative review article's goals are to outline the benefits of intermittent calorie restriction while accounting for any of its potential limitations and pinpoint any knowledge gaps that may exist.

## Introduction and background

**Figure 1:**
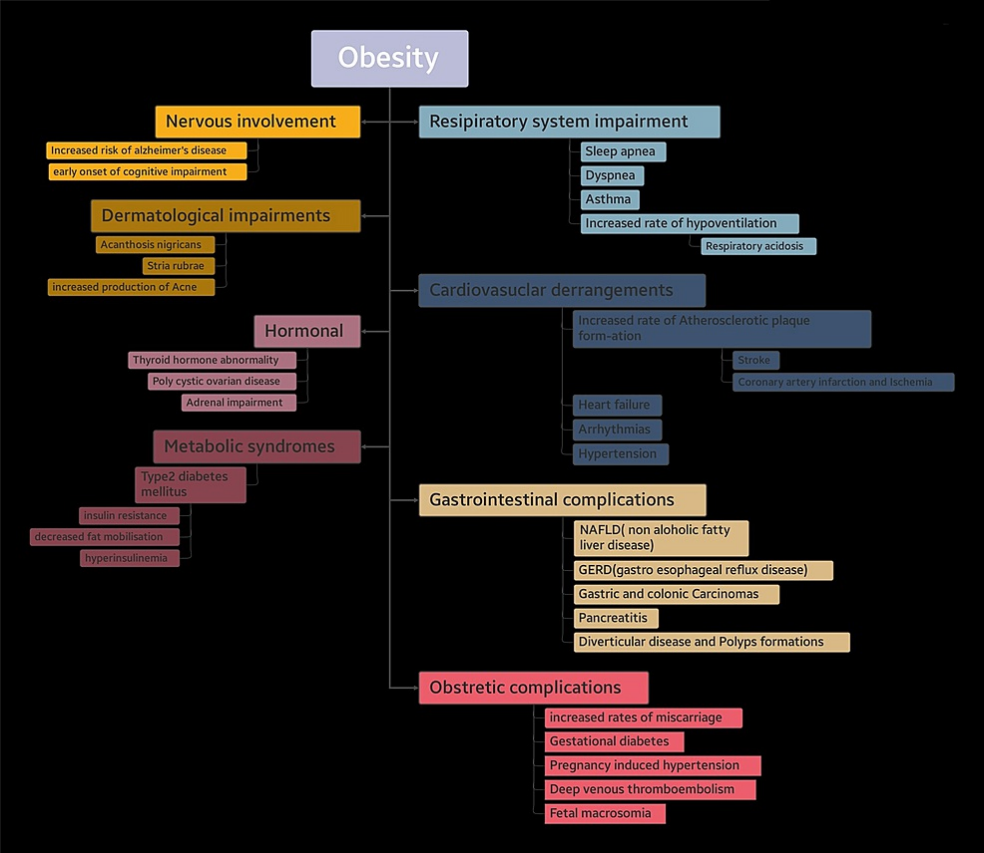
Consequences of obesity in multiple organ systems NFALD, non-alcoholic fatty liver disease; GERD, gastroesophageal reflux disease.

Obesity is one of the major debilitating factors to public health in this twenty-second century. It has almost tripled in amount since the late twentieth century so by 2016, around 1.9 billion adults were obese and it was roughly estimated that about one out of every three people was either obese or overweight/borderline obese. Obesity is typically described as an abnormal buildup of fat in the adipose tissues that interferes with the normal function of tissues [1]. Obesity is measured by evaluation of the body mass index (BMI); a BMI over 24 and below 30 kilograms per meter square is described as overweight and BMI over 30 kilograms per meter square is categorized as obesity as shown in Table 1 [2]. There are other methods like measurement of waist circumference and DEXA (dual-energy x-ray absorptiometry) scan for evaluation of peripheral fat and lean tissue distribution. The most common cause of obesity is overnutrition or a large calorie surplus. There are other factors influencing the rate of weight gain such as the amount of physical activity or lack thereof and nutritional status. There are certain pathologies that can also mimic obesity, such as hypothyroidism, which would influence the thyroid and thyroid-stimulating hormone levels leading to excessive fat accumulation; other such conditions are Cushing’s disease and polycystic ovarian syndrome. Controlling this alarming increase in obesity is crucial since an obese person is more likely to develop a number of comorbid conditions, including atherosclerosis, impaired glucose tolerance, hypertensive symptoms, stroke, and respiratory diseases, as seen in Figure 1 [3]. The Framingham Heart Study described a correlation between obesity and hypertension very well. A recent cross-sectional study that was conducted in Spain to ascertain the prevalence of obesity as well as the overweight population within the sample group of employees and measuring the correlation with diabetes mellitus, hypertension, and metabolic syndromes found that out of the sample population of 23,729 people, the prevalence of overweight was 38.6% and the prevalence of obesity was 18.4%, while the prevalence of diabetes mellitus was 7.6%, hypertension was around 20%, lipid profile abnormality was 31%, and other metabolic abnormalities were 7.5%, which further proved that there was a significant correlation between overweight and obese population and the prevalence of diabetes, hypertension, dyslipidemia, and other metabolic syndromes [4].

**Table 1:** Classification of BMI BMI, body mass index.

BMI (kg/m^2^)	Classification
Below 18.5	Underweight
18.5-24.9	Normal range
25-29.9	Overweight
Over 30	Obese
30-34.9	Grade 1 obesity
35-39.9	Grade 2 obesity
Over 40	Grade 3 obesity (morbidly obese)

This rise in obesity created a requirement for certain forms of intervention for its management depending on the patient. This can vary from medical management to certain surgical procedures such as liposuction and other bariatric surgeries for patients with pathological obesity. Among these methods of weight control came along a much more popular and widely socially accepted strategy of weight control in the form of dietary modifications. These dietary modifications grew in popularity as they were non-interventional for most of the time, hence are better accepted by the masses. Some of these common diet modifications are based on either nutritional restriction like the ketogenic diet where the major bulk of the daily calorie intake is through the consumption of fats and rest through protein with carbohydrates being as little as possible in the diet, and the paleo diet, which recreates the diet humans used to have in the paleolithic era; these were few of the very common nutritional basis of dietary modification, and the other is the topic in focus here, that is, time-restricted feeding (TRF), which in common terms is also called as intermittent fasting (IF).

IF/intermittent caloric restriction is a form of restriction of feeding into a smaller time window and a larger fasting window. The duration of fasting ranges from restriction for certain hours and feeding in the remaining days to fasting for alternate days, creating a few short pockets of calorie deficit while remaining in a short deficit from the baseline calorie requirement. This is abstained from the harmful effects of prolonged fasting or long-duration caloric restriction. For the betterment of body tissue make-up and general health, a range of regimens known as IF alter the time of meal intervals by utilizing brief fasts [5]. There have been studies proving that IF or a form of calorie restriction over the long term has been very important in maintaining optimum metabolic health and increasing longevity. Based on the interval of feeding, IF is categorized into four distinct groups: 1. Alternate-day fasting (ADF): this method contains a day with complete abstinence from food followed by a day of feeding, creating a window of 24 hours fast between subsequent feeds; 2. modified IF regimen: this method consists of eating in a calorie deficit of about 25% below the maintenance calorie requirement for a majority of the week and then creating a short window of feeding up to the limit of maintenance calories for a day or two, and the most popular regimen among this method is the 5:2 method, which requires five days pf partial fasting with eating below the maintenance and two days of eating at the maintenance calories; 3. time-restricted calorie consumption: it is the widely recognized method of IF, and this method allows participants to consume food as per their desire in a very specific short time frame creating a fasting period on a daily basis; few of the common time frames for such are the 16:8, 14:10, and 12:12 time frames where fasting is done throughout the longer time frame and feeding is done in the short window; 4. religious fasting: a variety of fasting regimen exists across cultures which dictates a specific form of fasting, and example of such fasting is Ramadan fasting as seen in Table 2 [6]. In 1963, Randel gave the fast-fed cycle theory of energy anabolism and catabolism during the period of fast and feeding [7,8]. The mechanism of IF in this case is it affects the fast-fed cycle and the circadian rhythmicity of the free fatty acids metabolism. Circadian rhythm is the most important factor for hormone synthesis and secretion as well as for gene expressions and is also responsible for neuro-hormonal regulation of weight. Overnight fasting is linked with a fall in blood lipid profile, decreased satiety, and mobilization of visceral fat through growth hormone regulation during the night, causing a further increase in hepatic gluconeogenesis [9]. Reduced carbon dioxide production versus oxygen consumption suggests improved metabolic flexibility and efficiency of fatty acid and ketone body metabolism and energy synthesis. This leads to a metabolic change from the consumption of glucose as a primary energy medium to the consumption of ketone bodies [10]. These ketone bodies are not only the energy source during the periods of fasting but also major signaling molecules; these ketone bodies further influence various molecules influencing aging [11-13].

**Table 2:** Types of intermittent fasting with their feeding and fasting windows TRF, time-restricted feeding; eTRF, early time-restricted feeding.

Types of intermittent fasting	Definition	Fasting window	Feeding window
Alternate-day fasting	Fasting a whole day followed by a day of feeding (in an energy deficit).	24 hours	24 hours
Modified intermittent fasting	Eating in an energy deficit for majority of the week followed by eating at baseline level for 2-3 days. An example of this is the 5:2 routine.	24 hours/day for 5 days (eating 25% below baseline)	24 hours for 2 days (eating at a baseline level)
Time-restricted feeding (eTRF)	Eating in a short energy deficit over a short feeding window followed by a longer feeding period.	16/14/12 hours	8//10/12 hours
	Modified form of TRF aligning with circadian rhythm.		
Religious fasting	Culturally dictated specific form of fasting. Ramadan fasting is an example of this type.	After sunrise (for Ramadan fasting)	At the time of sunrise (for Ramadan fasting)

## Review

Intermittent fasting and weight loss

Theoretically, intermittent calorie restriction has many benefits regarding weight loss and increasing insulin sensitivity. A study was done by Harvie M et al. (2013) [14] for the purpose of comparing the claim that intermittent calorie cutback is prevailing to daily calorie cutback in terms of reduction of weight and insulin sensitivity. They tested two intermittent regimens: one that had carbohydrate restriction (IECR) regimens, and one that followed carbohydrate restriction along with protein and fat (PF) monitoring (IECR + PF) [14]. One hundred and fifteen overweight women between the ages ranging from young adults to older age groups with a familial history of breast cancer were randomly assigned to receive a total energy restriction (ER) of 25%, either as an IECR that included 2500 to 2700 kJ per day, less than 40 grams of carbohydrates each day for two days every week, or a 25% daily energy restriction (DER) that was roughly 6000 kJ/day for seven days, or a combined regimen with restriction along with protein and fat surveillance for a three-month period. The IECR diet and the IECR+PF diet were found to lower insulin resistance. When compared to the DER diet, the reductions achieved with the IECR diets were noticeably better. In comparison to the DER group, both IECR groups (IECR and IECr+PF) showed a significant decrease in body fat. They were successful in demonstrating the advantages of intermittent calorie restriction diets in weight loss and decrease in insulin sensitivity over a shorter time frame, as it was discovered that even during the maintenance week/phase of the IECR and IECR+PF diet, the decrease in insulin resistance and weight found during the energy reduction weeks/phase was still present [14].

Another such study demonstrating the benefits of a short-term cycle of IF was done by Byrne NM et al. (2017) [15]; it was suggested that a two-week block of energy balance might be created by combining an optimal intermittent energy reduction cycle with ER phases of the same length. This would provide enough time to reduce adaptive thermogenesis. In this study, the researchers wanted to know if intermittent ER was more efficient at helping people lose body weight than continuous ER. In this randomized controlled trial for the selection of participants, 51 participants with obesity were randomized for the duration of 16 weeks of fasting alternating with 14 weeks of sustained energy intake, combining to a 30 weeks total. After completing a four-week baseline phase, the protocol was begun with 47 participants. Energy intake for both groups during the calorie restriction phase was 67% of what was needed to maintain their weight. The target of the study was to calculate the total weight, mass occupied by fat, fat-free mass, and resting energy dissipation during the whole period of the study. The results of this study found that for the 19 subjects in the CON (continuous) group and 17 subjects in the INT (intermittent) group who completed the procedure as per their decided regimen, weight loss was far superior for INT which was around 5 kg more. INT also showed more reduction in fat mass, that is around 4 kg, but the fat-free mass reduction was not very different from CON, which was less than 0.5 kg. The mean weight alteration during the 14 weeks in the INT groups was not very large. The decrease in absolute resting energy utilization was almost the same as before among the groups, but after compensating for alteration in the body tissue make-up, it was quite significantly reduced in the INT group. The study discovered that intermittent exercise lowered compensatory metabolic responses, which enhanced the effectiveness of weight reduction. Intermittent restriction of energy consumption with energy balance periods is also shown to reduce weight loss as well as fat loss [15]. 

Despite its proposed benefits, intermittent calorie restriction also has some drawbacks; these drawbacks come in the form of defaulting in compliance and nonadherence to the program. Efficacy, tolerance, and safety of the ADF variant of intermittent calorie restriction were measured in another trial, which was conducted by Catenacci et al. [16]. This trial sought to compare and assess the changes in weight, body fat distribution, and lean mass composition, lipids, and effects on insulin resistance to those brought about by a generalized weight-loss diet that combined a medium daily caloric restriction with ADF in order to assess the safety and tolerability of ADF method of intermittent calorie restriction. The participants selected were adults who had obesity on the basis of their BMI and were between the ages of 18 years and 55 years of age. The participants were then sorted in a randomized pattern into the calorie restriction group with 12 participants in it which followed a daily deficit in their energy consumption and the ADF group which had 14 participants for eight weeks. The outcomes of the study were first evaluated at the end of the eight-week protocol and then again after 24 weeks of unmonitored follow-up. The findings of this study showed that ADF had no negative health consequences, and over 90% of the population completed the eight-week ADF procedure. When weight, body composition, lipids, and insulin sensitivity were taken into account, the ADF group obtained a higher calorie deficit at the end of eight weeks, but there were no discernible intergroup differences. The differences between losing weight and gaining it after 24 weeks of follow-up without any supervision came up to be insignificant, although deviation from baseline in percentages of lean tissue content and fat tissue content was higher in favor of ADF. They demonstrated the safety and acceptability of ADF as a weight-loss strategy. At the end of eight weeks, ADF produced comparable improvements in weight, morphological improvement of body tissue composition, reduction of risk of vascular pathologies, and improved insulin resistance [16].

Can intermittent fasting have cardioprotective features?

It is very evident that coronary artery disease is one of the common and deadlier outcomes of obesity [17], and the effect of calorie restriction on cardioprotection is also very well studied [18]; that being said, it was important to study if intermittent calorie restriction can provide a cardiovascular protective effect along with above-elaborated weight control functions. A study by Varady KA et al. (2009) [19] assessed how well ADF changes the risk factors of important coronary arterial disorders in particularly obese individuals and contrasted the amount of weight reduction that can be precipitated by ADF during a period of monitored food consumption against a period of unregulated food consumption while receiving nutritional advice. Sixteen obese participants were selected, and they completed a 10-week trial, which consisted of three stages: control phase, weight reduction/ADF monitored diet consumption stage, and fasting but with ad libitum dietary intake. The outcome of this trial was no particular dip in dietary adherence by the participants; in fact, the dietary adherence remained high and over 80% during the monitored dietary intake phase, and the ad libitum food intake phase had even more adherence, which was about 89%. No fluctuations in the rate of weight loss can be seen throughout the controlled food intake as well as in ad libitum/self-selected food intake phases. The weight of the subjects was found to be decreased by an average of 5.5 kg after continued adherence to the diet. Body fat also fell by 3%. After eight weeks of ADF, there was an improvement in the lipid profile. High-density lipoprotein showed no significant alterations. The blood pressure decreased and systolic BP saw a decrease of 18 mm Hg. All of these study results support the idea that ADF can assist obese and overweight people in losing weight and body fat and lower their risk of developing cardiovascular disorders [19].

Promising prospects of intermittent fasting combined with exercise

Although it has long been recognized that both aerobic and anaerobic exercise are essential for weight loss, aerobic exercise is still the most effective method for improving anthropometric measurements, including body weight and visceral fat constitution [20]. Regardless, it is a universal fact that an optimal combination of aerobic and aerobic exercise holds great importance for the control of obesity [21]. This fact raises a question: if intermittent calorie restriction was followed along with monitored exercise, will it provide even better results in controlling obesity, dyslipidemia, and cardiovascular diseases? Looking at one such study trying to answer this question, Bhutani S et al. (2013) [22] conducted research on how alternate-day ER, when followed along with aerobic patterns of exercise, affected obesity and cardiac disease risk factors. For this study, 83 participants were selected and placed into four categories based on the intervention they were given; the first group consisted of obese males and females having a sedentary lifestyle to be placed in the combined regimen group (ADF plus exercise) having 18 participants and then compared to the second ADF-only group having 25 participants, a third exercise-only group with 24 participants, and fourth a control group with 16 participants. The duration of the study was 12 weeks. The important criteria for inclusion of participants were as follows: adults to early old age individuals within obesity class 1 and obesity class 2; the weight of the participants should not be fluctuating in either direction for a few months prior to the initiation of the trials; participants should not have diabetes; participants must not have a history of heart disease; participants should be habitual in performing light physical activity; participants must not be smokers, and they should not have a history of weight-altering medical interventions, and lipid-profile-altering or glucose-mobilizing medications. The combination and ADF groups were given a dietary intervention, which was composed of two phases: 1) four weeks of monitored feeding phase and 2) eight weeks of ad libitum feeding phase. During the monitored feeding phase from the first week to the fourth week, participants consumed one-fourth of their minimum calorie requirement on the specified day set for fasting and consumed food without monitored dietary choices on specified days set for feeding; both of these days lasted for 24 hours. The minimum energy demands were approximated by the Mifflin equation [23]. Both exercise and combination groups underwent a well-watched exercise intervention. For 12 weeks, these participants engaged three times a week in monitored moderate-intensity activity. The physical activities were of aerobic form. Although there were 83 participants selected in total, there were only 64 participants who completed the trial, whereas there were 16 participants who completed the trials in each group. The number of dropouts was the most in the alternate fasting group with nine dropouts, followed by the exercise-only group with eight dropouts when compared to the combination with two dropouts and no dropouts in the control group. The study found that body weight decreased by 6 kg in the combined group, 3 kg in the ADF group, and 1 kg in the exercise-only group. Lean body mass remained constant in the combination group, whereas fat and other anthropometric measurements both decreased. Only the combination group showed an LDL decrease of 12% and an HDL increase of 18%. Only the combination group experienced a decrease in the proportion of tiny HDL particles. All of these results convinced us that ADF and exercise alone were not sufficient for body weight loss, but that when IF is combined with a structured exercise program, all the results indicate a positive health spectrum and prove the hypothesis of this study [21]. Aerobic exercise along with IF has been shown to have its benefits, but what about the interaction of resistance training and IF? To answer this question, the trial by Moro T et al. (2016) [24] was conducted to determine the impact of a common form of IF - the 16/8 time-restricted eating type of IF - on male resistance-trained athletes' basal metabolic rate, maximum strength, body composition, and cardiovascular disease risk factors. Thirty-four male participants who engaged in resistance exercise were placed in a randomized pattern in either the TRF groups or the control in this study. TRF participants were made to eat all of their daily caloric requirements in an eight-hour timeframe, with their caloric intake being split into three meals: the first meal was to be consumed at 1 pm, and nothing was to be consumed after the last meal. The subjects in the control group also ingested their daily energy requirements spaced out among three meals without any time restriction. Calorie and macronutrient distributions were identical between groups. Tests were conducted before and after the generalized resistance training program and the specified nutrition regimen for eight weeks. The DEXA scan was used to measure the anthropometric parameters, testosterone was profiled, pancreatic and adipose tissue-influencing hormone, thyroid-influencing hormone, inflammatory markers, and lipid profile tests were done. The maximum strength of the compound lifts, resting energy consumption, and respiratory ratio were also assessed. After eight weeks of testing, the two-way ANOVA (analysis of variance) revealed that time-restricted eating reduced fat mass when compared to a regular diet, but neither group's lean muscle mass, arm, and thigh muscle content, or maximum strength changed. Significant reductions in both testosterone and insulin-like growth factor 1 were seen in the TRF but not in the normal diet group. Adiponectin increased in the TRF while total leptin decreased. T3 was reduced in TRF, but the thyroid-stimulating hormone remained unchanged. No significant alterations in the lipid profile were seen. Resting energy expenditure remained the same, but the respiratory ratio fell substantially in the TRF group. Results show that resistance training improves several biomarkers associated with cardiovascular health, lowers fat mass, and preserves muscle mass in men who have undergone time-regulated feeding (IF) with an 8-hour meal window [24,25].

Intermittent fasting and its influence on metabolism

Multiple studies and trials have demonstrated the pros of IF for physiologically improved anthropometry and fat tissue and lean tissue morphologies. However, for a long time, it was believed that IF only had an impact on controlling obesity because it produced a consistent and sustained calorie deficit. Through various recent studies, it has been found that IF also modifies the enzymes and hormones and has an overall metabolic alteration. The circadian rhythm is a pattern that affects the body's metabolic rate and synchronizes biological processes with predictable and adaptive environmental patterns to promote optimal performance and health [26,27]. IF/calorie restriction, particularly when the food window is early in the morning or before midday, has been linked to lower body weight and fat mass as well as better blood pressure and insulin sensitivity, according to a number of recent research [24,28]. The impact of IF on metabolic processes, whether in health or sickness, should not be ignored because several implications of TRF on hypothalamic, pituitary, and both hormones have previously been reported via diverse research [29-32]. This raises a certain question if IF can modify the makers for diabetic and pre-diabetic patients. In a randomized trial conducted by Sutton EF et al. (2018) [33], this study also becomes the first trial of early TRF, a type of IF that revolves around a circadian rhythm in which feeding occurs earlier to align the metabolism with the circadian rhythm, to see if IF has its benefits independent of weight loss by feeding participants at baseline maintenance calories. For eTRF, the feeding window is for 6 hours only, and the last meal should be taken before 3 pm. Men who were pre-diabetics served as the study's subjects, and they were placed after randomization to either an eTRF regimen or a control schedule for five weeks before switching schedules. According to the study's findings, eTRF enhances insulin sensitivity, cell responsiveness, blood pressure regulation, oxidative stress reduction, and appetite. They suggested that eTRF enhances several cardiac and metabolic health factors and that IF's benefits extend beyond weight reduction [33].

Does intermittent fasting have benefits on a long-duration basis?

The benefits of the short term usually between 12 and 18 weeks of IF regarding weight loss and even management of various diabetic parameters have been very evidently shown through the studies [14,19,22,34]. Arnason et al. (2017) [34] investigated the biological-chemical changes occurring in a shorter time frame caused due to IF of TRF type in 10 individuals having type 2 diabetes mellitus taking metformin, an oral anti-diabetic. The study's findings were as follows: significant weight loss, a decrease in BMI, and at-target morning glucose were all seen after short-term IF. Results also indicated that lengthening the fasting period reduced fasting blood sugar levels. However, neither insulin resistance nor the inflammatory markers c-reactive protein stabilized during the IF phase. These results support IF's short-term advantages once more. It was challenging to assess the advantages of long-term intermittent calorie restriction because there is a lack of long-term IF studies. However, a trial by Trepanowski JF et al. (2018) [35] proceeded to evaluate the claims that ADF type produces superior improvements in overall body tissue distribution and composition, and enhances the adipokine profile when compared to the continuous calorie restriction pattern. To test this claim, a secondary analysis of a randomized controlled trial was conducted. Changes in the morphological, as well as imaging parameters of body tissue composition and the adipokine assay, were compared by these methods. This study was carried out over the period of 6 months/24 weeks where 100 obese or overweight participants were divided into three groups: 1) ADF, rotating every alternate day between consuming one-fourth or slightly more than the baseline of total caloric demand; 2) calorie restriction (CR), consuming three-fourth of caloric demand each day; 3) the control group. The results at the end of 24 weeks were as follows: the VAT:SAT (visceral to subcutaneous abdominal fat) ratio remained the same in all groups. However, the ratio of fat-free mass to the total body mass did rise in both ADF and calorie restriction, but when compared to the control group, the interventions performed in both groups were identical. There was a decrease in leptin found in circulation in both ADF and CR groups. Adiponectin and inflammation markers did not change in either of the groups. Following a 24-week trial, ADF was shown to increase fat-free mass:total body mass, decrease VAT:SAT ratio, and fall in leptin levels [35]. This research backs up the long-term advantages of ADF.

Potential drawbacks of intermittent fasting

Although the benefits of IF are tremendous, it is still important to look at any potential drawbacks of IF. One of the most commonly imaginable drawbacks of this particular dietary regime is a lack of adherence to the diet, but in a more realistic scenario, it is very much unlike so. According to a study done by Dorothea K (2019) [36] to assess the adherence to IF and its impact on particularly abdominal obesity, in this study, 40 subjects with abdominal obesity were selected, who were then made to follow the 16-8 pattern of IF for three months of duration. The results found were, out of the 40 participants, 23% of the trial population found adherence to the diet extremely easy, 28% found it neither to be very challenging nor extremely easy, and only 10% found IF to be challenging enough that adhering to diet was an uphill task for them. Among these 40 participants, a majority, about more than 50%, showed complete adherence to the diet and never deviated from it even once; around 20% showed a tendency to deviate once a week, 10% deviated more than once a week, and only one participant deviated daily from the regimen. A majority of the test population, little more than 70%, found IF to be extremely beneficial for weight loss. About 20% found it had neither a positive nor a negative impact on the program and only one participant found the regimen to have a negative impact. This study determined that there is an overall positive outcome when it comes to ease of adherence to IF; along with this, IF also showed a positive impact on weight control over 60% of the test population [36]. Another study was done by Hoddy KK (2015) [37] particularly to assess the safety of IF, where a total of 74 participants with obesity were selected for three weeks of ADF form of IF. Out of these 74 participants, only 56 participants successfully completed eight weeks of IF, and there were many reasons for dropout like difficulty with adjusting to the diet, conflicts with schedules, and other personal reasons. On the positive side, there was a positive decrement in body weight and fat mass, and also some decrement in lean mass. Some of the recorded adverse effects were constipation in 17% of the subjects, 2% of participants also experienced water retention, 14% of participants reported foul breath during the period of eight weeks which later doubled to 28% by the end of 8 weeks, less than 10% of the subjects noted the difficulty in staying asleep, while some also complained of generalized weakness. However, there was a slight decrease in depression and binge-eating disorder from the baseline and a slight overall increase in the positive body image of the subjects. This study also helps us solidify the remark that for IF the risks of any potential side effects are quite minuscule to outweigh the rewards of the process. But few in-depth studies on the long-term effects of IF are required to create more solid evidence against IF if they exist [37].

## Conclusions

Due to its severe and debilitating comorbidities, obesity is a worldwide health burden and a major source of cause of death and disability. This calls for an immediate and unique management technique that can be widely accepted by the public. Filling this spot is IF or intermittent caloric or ER; the principle of this form of calorie restriction was to create a fixed small gap for eating and then create a large window for fasting allowing the body to tap into its adipose stores and its glycogen stores for energy leading to decrease in body fat and body weight and control of obesity; along with this, the article also narrated the importance of maintaining the diabetic parameters in the diabetic and the pre-diabetic patients. An argument can also be made for potential ill effects of IF, ranging from its effect on psychiatric disorders such as depressive disorder and binge-eating disorders to much more systemic effects like gastrointestinal disorders such as constipation and water retention, but time and time again studies have confirmed that the benefits of IF greatly outweigh the drawbacks of it as long as the compliance to the diet is there. A large number of short-term studies elicit the value of IF in a short time frame; however, such positive claims for long-term IF are still quite hazy due to the lack of long-term IF studies. This creates a scope for long-term IF trials and to study their benefits.
